# A specific method for qualitative medical research: the IPSE (Inductive Process to analyze the Structure of lived Experience) approach

**DOI:** 10.1186/s12874-020-01099-4

**Published:** 2020-08-26

**Authors:** Jordan Sibeoni, Laurence Verneuil, Emilie Manolios, Anne Révah-Levy

**Affiliations:** 1 Service Universitaire de Psychiatrie de l’Adolescent, Argenteuil Hospital Centre, 69 Rue du Lieutenant Colonel Prud’hon, 95107 ARGENTEUIL Cedex, France; 2 ECSTRRA Team, UMR-1153, Inserm, Université de Paris, F-75010 Paris, France; 3 Service de Psychologie et Psychiatrie de Liaison et d’Urgences, Hôpital Européen Georges Pompidou AP-HP, Hôpitaux Universitaires Paris Ouest, Paris, France

**Keywords:** Qualitative research, Patient-reported outcomes, Research methodology, Care

## Abstract

**Background:**

This paper reports the construction and use of a specific method for qualitative medical research: The *Inductive Process to Analyze the Structure of lived Experience (IPSE),* an inductive and phenomenological approach designed to gain the closest access possible to the patients’ experience and to produce concrete recommendations for improving care. This paper describes this innovative method.

**Methods:**

IPSE has five steps: 1) set up a research group, 2) ensure the originality of the research, 3) organize recruitment and sampling intended to optimize exemplarity, 4) collect data that enable entry into the subjects’ experience, and 5) analyze the data. This final stage is composed of one individual descriptive phase, followed by two group phases: i) structure the experience, and ii) translate the findings into concrete proposals that make a difference in care.

**Results:**

This innovative method has provided original findings that have opened up new avenues of research and have important practical implications, including (1) the development of patient-reported outcomes, (2) clinical recommendations concerning assessment and treatment, (3) innovative ways to improve communication between patients and doctors, and (4) new insights for medical pedagogy.

**Conclusions:**

IPSE is a qualitative method specifically developed for clinical medical research to reach concrete proposals, easily combined with quantitative research within a mixed-method study design and then directly integrated within evidence-based medicine.

## Background

### The need for a new qualitative method conceptualized by physicians

As the role of patients in their own medical management radically evolves, more collaborative practices that consider the patient’s perspective in this process are progressively replacing the older approach of paternalistic medicine [1]. Patients’ preferences, choices, and needs have been placed at the core of treatment. This idea relies on a paradigm shift that places the patient’s lived experience at the center of the care process. That is, patients are now considered to be the expert on their own lived experience; and their voices must be heard to enable the achievement of a more person-centered medicine. This paradigm shift is illustrated by the development of concepts such as patient experts [2], patient partners [3], and peer-support workers in psychiatry care [4], but also by the evolution of the principles of evidence-based medicine (EBM). The concept of EBM emerged in the 1980s with the aim of rationalizing medical practices and hierarchizing the medical literature. This concept first relied only on (i) external clinical data (that is, results from randomized control trials and meta-analysis), with (ii) medical expertise subsequently included, and most recently, (iii) patients’ preferences added [5]. EBM considers, nowadays, that the best-informed medical decision is the one at the intersection of a Venn diagram composed by these three circles. This paradigm shift is also illustrated by the development of new concepts intended to capture patients’ preferences better: patient-reported outcomes (PROs) and patient-reported outcome measures (PROMs). According to the FDA-NIH Biomarker-Working-Group glossary definition, a PRO is “a measurement based on a report that comes directly from the patient, about the status of a patient’s health condition without amendments or interpretations of the patient’s response by a clinician or anyone else” [6]. Today, clinical trials use PROs/PROMs increasingly often [7]. They are essential outcome measures, demanded by health authorities and regulatory agencies, and useful for physicians, patients, and health policy-makers. In this new era of person-centered medicine, many authors have concluded that an initial phase of qualitative research is needed early in the construction of all PRO tools to explore patients’ experiences [8–10]. The PRO Good Research Practices Task Force of the International Society for Pharmacoeconomics and Outcomes Research (IPSOR) [10, 11] has also published suggestions for conducting qualitative studies intended to support the content validity of PRO instruments.

Qualitative health research (QHR) is a relatively recent field, covering a broad area — from the description of illness experience to the sociocultural organization of health care — and using a myriad of qualitative methods coming from other theoretical fields, mostly social sciences: sociology (i.e., symbolic interactionism with grounded theory [12], ethnography [13]), psychology (i.e. phenomenological psychology [14–17]), case study, narrative, and linguistics [18, 19]. Other qualitative methods have been developed specifically for QHR in applied disciplines, mainly by and for nursing sciences [20, 21], and in primary care [22], focusing on specific issues (e.g. experience of specific illness, of caring, of help seeking). Qualitative research is now booming in biomedical clinical studies [23] in many medical specialties, aimed at obtaining an in-depth understanding of phenomena, directly from the perspective of the people experiencing them. These studies have either applied a qualitative method from another field, or relied exclusively on a thematic analysis approach intended only to structure how the data are analyzed and the results presented [24]. According to Morse, one goal of QHR is to “bridge the gap between scientific evidence and clinical practice” [20]. This is already the case at a “review” level with the work of the “Cochrane Qualitative & Implementation Methods Group” for the dissemination and incorporation of qualitative results in systematic reviews, that is, *qualitative evidence synthesis* [25]. But, so far, there has been no medical qualitative research method specifically tailored to produce rigorous data from the lived experiences of both patients and physicians to directly inform EBM. For instance, there are no specific qualitative methods to explore the perceived efficacy of a treatment to determine efficacy criteria relevant for patients themselves. Medical research should expect qualitative studies to produce knowledge with the potential to improve patients’ care and lives, and not simply conceptual knowledge, that is, knowledge for its own sake, as qualitative methods from the social sciences produce [20]. Within nursing research, Thorne has developed “interpretive description”, an inductive qualitative method with roots in phenomenology, ethnography, and grounded theory but which endeavors not to theorize the results but rather to offer practical outcomes for nurses’ daily practices [21]. Although applicable to other areas of health, including clinical medical practice [26, 27], interpretive description does not focus on the lived experience of the stakeholders but rather on contextualizing illnesses in multiple domains (e.g. experiential, spiritual, political, cultural, etc.).

Our group, which has worked more than a decade on the analysis, dissemination, and use of qualitative methods in medicine, has developed expertise in their use for exploring complex questions around the experience of diseases and their treatment [28–30]. We consider that physicians have specific concerns and that their medical training and professional experience enable them to contribute to the field of qualitative medical research differently than nurses and other healthcare professionals do. Thus, as both medical doctors and experienced qualitative researchers, we have become convinced of the need for a new qualitative method designed by physicians for addressing specific issues in clinical medical research.

We advocate that this new method should meet several criteria:

1. To thoroughly capture the lived experience of patients and other stakeholders: how they live their disease and its treatment and how they recount it [31], not as an end but a means, to connect experiential knowledge with physicians’ medical knowledge and to develop concrete proposals for improving the health care pathway, treatment, and clinical research.
2. To be completely structured to allow a group of physicians, after receiving appropriate training, to conduct rigorous, systematic qualitative medical studies, that is, with all stages of the design clearly described and operationalized.
3. To use vocabulary and concepts that make qualitative medical research accessible and meaningful for physicians and for administrative and policy-making bodies
4. To directly involve patients within the research process
5. To integrate qualitative medical research within EBM.

Since none of the existing qualitative methods meet all these criteria, we decided to progressively develop our own: *Inductive Process to analyze the Structure of lived Experience* (IPSE).

### IPSE: theoretical backgrounds

Some qualitative methods have been suggested in medical research, such as qualitative theory-development studies or qualitative elicitation research within a cognitive model [32]. These are based on directive, task-oriented interviews that we think excessively restrict the direction of the conversations for both participants and interviewers. We want instead to keep the research open to what the participants’ narratives of the experience can add, to allow them to share what they have lived. We strongly believe that truly taking their experience in dealing with an illness or its treatment into account requires letting them recount it freely, as they want to and see fit. The exploration of the lived experience is for us the core of what qualitative methodology can contribute to medical research. To do so, the method must fit into the constructivist paradigm [33, 34] and be informed by a phenomenological approach [14], but without overly theorizing the underlying epistemological and philosophical knowledge, which would impede its practicability.

Lived experience can be defined as personal knowledge of the world gained through direct participation and involvement in the event or phenomenon. Lived experience refers to human activities that are immediate, situated and daily, which are lived without thinking about or paying attention to them (pre-reflexive experience) [35].

Constructivism comes from the work of the philosopher Immanuel Kant [36]. It considers that knowledge emerges from a human process of construction. As a research paradigm, constructivism conceives knowledge as a shared construction built on the encounter between researchers and research participants [33, 34]. Many qualitative methods, including ethnographic, narrative, and phenomenological, fit into this paradigm, —or have been adapted to it, such as Charmaz’s adaptation of Grounded Theory from a constructivist perspective [37]. IPSE fits epistemologically into a constructivist paradigm as we postulate that the production of knowledge relies on three elements: (i) subjectivity as a space for constructing human reality, (ii) intersubjectivity as a strategy for accessing valid knowledge of human reality, and (iii) understanding that human reality takes place in daily life. These elements underlie the strengths of this method, which is characterized by flexibility in the progressive construction of the object under study, constantly adjusted to the characteristics and complexity of human phenomena, and always takes the subjectivity of the researchers and the participants into account while combining several techniques of data collection and analysis.

Phenomenology literally means the *study of what appears*. At the very beginning of the twentieth century, phenomenology became the name of a philosophical current founded by Husserl [38]. He aimed to study how objects appear to the subject’s consciousness and to describe the essence of a phenomenon not by describing the object as it exists but by describing the experience of the subject. Phenomenology can be descriptive or interpretive, that is, associated with hermeneutics (science of interpretation) [39]. Within qualitative research, phenomenological approaches seek to capture the lived experience of a subject about a phenomenon, to understand how this phenomenon appears in the individual’s conscious experience. In the field of phenomenology, experience is conceived as uniquely perspectival, embodied, and situated. Phenomenological approaches are particularly relevant for conducting research on experiences, thoughts, imagination, intentions, desires or volition. There are many phenomenological qualitative approaches, coming mainly from the field of psychology, either descriptive (Giorgi’s descriptive phenomenological approach [14], its adaptation by Colaizzi [40], and Moustakas’s heuristic method [16]) or interpretative/hermeneutic (interpretative phenomenological analysis (IPA) and Van Mannen’s approach) [15, 17]. Many of them use a theoretical vocabulary and philosophical concepts that are not easily accessible for physicians. Our early work used well-known qualitative phenomenological approaches: one descriptive, that is, Colaizzi’s method [41] and one hermeneutic: IPA [42–48].

Recently, philosophers working in the field of phenomenology have criticized some of these methods. Zahavi wrote about the approaches of IPA and Van Mannen that “qualitative health researchers interested in phenomenology should look elsewhere for theoretical inspiration and methodological guidance” [49]. As for Moustakas’s method, Appelbaum noted that, although it uses key phenomenological terms*, this* method is not phenomenological but rather grounded in a humanistic therapeutic perspective [50]. Moreover, hermeneutic approaches assume that human beings are always already engaged in interpretative meaning-making activities. They do not seek to capture the patients’ lived experience, but only the meaning they give to it. These aspects do not appear appropriate for application in medical research. In line with Thorne [21], we consider that the interpretative underpinning in qualitative medical research must be more pragmatic and focus on eliciting concrete proposals for improving treatment. A descriptive approach, that is, “develop [ing] a textural description, what the participants experienced, and a structural description, how they experienced it in terms of conditions, situations or context” [51] appeared to us more appropriate to integrate into EBM and PRO. However, the descriptive phenomenological approaches [14, 40] are mainly methods for analyzing qualitative data (i.e., interview transcripts) and not global research methods (methods structuring a systematic research process from A to Z). In particular, while they underline the need to collect data of first-person accounts of types of experience, they do not provide detailed instructions for the data collection process or study design. Furthermore, descriptive approaches consider access to the lived experience as the goal of the approach without any other more practical and concrete objectives or perspectives. As mentioned above, we consider that within qualitative medical research, lived experience should be considered a means rather than an end. All stages of IPSE are informed by a phenomenological descriptive approach, not only the analytical procedure, as each stage contributes in its own way to capture and describe the lived experience of the participants. At the same time, the objectives of IPSE differ from those of other phenomenological approaches used in qualitative health research: it seeks to improve the quality of care, by producing concrete measures (about treatment and care pathways) and to propose new avenues of research.

### The two cornerstones of IPSE

The choice of the name IPSE (Inductive Process to Analyze the Structure of lived Experience) underlines the method’s two cornerstones: the inductive process and the analysis of the structure of lived experience.

IPSE relies on an inductive process: the procedure is exploratory, and no research hypotheses are formulated before starting; rather, they emerge from the material, through methods designed to penetrate as far as possible into the participants’ lived experience. Because the data are collected and analyzed simultaneously, the analysis can affect the collection of the data, directly from the material, that is, the narrative of the participants’ lived experience [31]. The most exemplary inductive approach in qualitative research is Grounded Theory [12], in which the researchers must suspend their relations with previous theories and limit their review of the literature, so that they can be fully attentive to the unexpected and the novel and can allow local theories to be emerge directly by the context and the material [52]. The IPSE inductive approach does not, however, imply disregarding either practical or theoretical medical knowledge when formulating research questions and objectives. The starting point of an IPSE study is always an unanswered question about the experience of individuals involved in medical care, unanswered questions by experienced physicians specializing in the topic. These specialized physicians are part of the research group and contribute to all the stages, including the definition of the areas to be explore in the data collection procedure. However, in line with the grounded theory approach [12], the physicians are not to share their knowledge with the qualitative researchers conducting interviews and analyzing data.

A qualitative researcher is his or her own instrument [53] and his or her preexisting knowledge and preconceptions (i.e. assumptions, values, interests, theories, beliefs, emotions, etc.) influence how data are collected, explored, analyzed, interpreted, and presented [54]. Usually, researchers using a qualitative phenomenological approach claim they perform *époché,* that is, that they “bracket” or set aside their preconceived knowledge and preconceptions of the phenomenon being researched [54]. Phenomenological philosophers, however, argue that this husserlian term has been misused and misinterpreted by qualitative researchers [55]. Using terms such as époché and reduction would, we think, confuse physicians and impede the accessibility of the IPSE method to physicians. We consider instead, along with other researchers such as Moustakas [16], that what matters is not to bracket the preconceptions but to identify and acknowledge, and make them explicit, through the act and work of reflexivity [56], which we will describe fully later. Only in this way can researchers avoid blind spots and cognitive biases, that is, the systematic errors in thinking that occur when people are processing and interpreting information-, especially confirmation bias, selection bias and the curse of knowledge [57, 58] that can hinder both access to the participants’ experiential knowledge and the discovery of new useful knowledge.

The analysis of a structure of lived experience is the main goal of our method. The descriptive phase of the analysis is inspired by Colaizzi’s method [40], but our approach is systematic and the structure of the experience is not an end but a means, since it is translated into concrete proposals for improvement in the health care pathway, for treatment, and for clinical research.

## Method

**Fig. 1:**
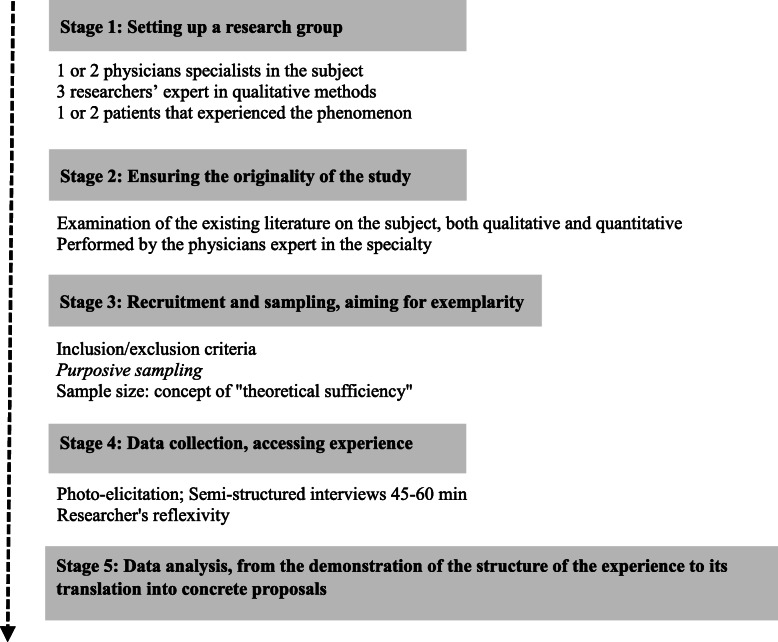
IPSE, a new method for qualitative research applied to clinical medical research, in 5 stages

The ISPE method has five stages that structure the entire research process (Fig. 1).

### Stage 1: setting up a research group

The research group always includes two physicians specializing in the topic, generally three researchers with expertise in qualitative methods, and, when possible one or two patients who experience the phenomenon under study.

In total an IPSE research group ideally contains five to seven members. This number is necessary to ensure that the study is rigorous and trustworthy.

- The two physicians specializing in the topic are necessary because they both perform the systematic review (stage 2) and ground all the research process into practical and theoretical medical knowledge without impeding the inductive process.
- The three qualitative researchers collect the data (stage 4) and analyze it (stage 5, descriptive individual phase). We consider that three are necessary to avoid confining the data collected and analyzed in the sole perspective of one researcher, or confronting only two opposing points of view.

As for the two patients: In both participatory [59] and heuristic approaches [16], participants are not viewed as study subjects but as co-researchers who are an integral part of the research process. IPSE strongly supports a more participatory approach in which service users should be directly involved in the qualitative medical research process [60], but with a different innovative strategy — to integrate directly, when possible and appropriate, into the research group one or two patients who have experienced the phenomenon or had the disease under study. This strategy follows the same principles as those underlying the use of peer-support worker in psychiatric departments to allow a more person-centered and recovery-focused approach [4]. Participating patients requires a short training on qualitative research in general and the IPSE approach in particular, which we provide in 3 days before the research starts. Integrating patients within the research group is not mandatory as it can sometimes be quite difficult to find such patients willing to be trained and participate. However, the research group must meet at least twice with “subjects of the experience” that is, patients other than the participants, in the same situation (via patient associations, for example) (i) at the very beginning of the research to develop a research question that really matters for the patients; and (ii) at the end to obtain feedback and validation of the results from the patients themselves.

This group oversees the entire research project, making all decisions collegially. We aim for heterogeneity in the group’s members, in terms of culture, knowledge, sex, age, occupation, and background. This diversity helps enrich the research at every stage, so that the results are more robust and relevant and not limited to a single perspective.

### Stage 2: ensuring the originality of the study

We follow the common principles of good practice in research, one of which is that a study must always begin by examining the existing qualitative and quantitative literature on the subject. Inductive approaches generally assume that to prevent interference by existing data, researchers beginning a qualitative study must not review the literature. On the other hand, there is little reason to replicate a qualitative study, since the importance of this type of research is measured by the novelty of the information it provides. It is thus important to avoid reinventing the wheel with each study, which would result in literature overloaded by similar but different or differently labeled concepts — what Morse describes as “theoretical congestion” [20]. Moreover, it is as important to ground the research in a rationale informed by medical literature, for example, by specifying or redefining the study objectives, as it is to remain attentive to the unexpected and novel [52] to produce original findings. This epistemological issue is known as *Meno’s paradox*, enunciated by Socrates in Plato’s Meno: **“**We cannot look either for what we know, nor for what we do not know; what we know because, as we know it, we do not need to look for it, what we do not know because we do not even know what to look for [61].”

To resolve this conundrum, we have developed an original group procedure: the two physicians who are experts in the topic conduct a systematic review of the qualitative and quantitative literature to confirm the study’s relevance and originality. To remain inductive and open to novelty, as mentioned above, the other group members have access to this review only after the data analysis has been completed. The tragedy of modern knowledge is, as Morin stated, that “the exponential increase in knowledge and references … stands in the way of reflecting on knowledge” [62]. It is therefore important that physicians share the minimum of necessary knowledge to inform the study without impeding it by the *curse of knowledge* [57].

### Stage 3: recruitment and sampling, aiming for exemplarity

After defining the research question, the group selects the study site or sites best able to optimize the feasibility of recruitment, depending on the study topic. It also defines the inclusion and exclusion criteria. In our method, sampling aims to attain exemplarity, that is, to select participants who, according to the research group (especially the physicians and the patients), have experienced quintessential, typical, or archetypal examples of the situation being studied. It thus uses *purposive sampling,* that is, selects the subjects likely to provide the most information about the phenomenon studied [63]. Unlike other recruitment strategies in qualitative research (i.e., homogeneous or convenience sampling), we are looking for a variety of exemplary situations by including participants who might enrich and add something new to what was previously found. The patients included might thus differ by sex, age, social and family status, degree of involvement, disease history, comorbidities, duration of treatment, and outcomes. This enables a broader understanding of the phenomenon under study. Because the analysis takes place simultaneously with the data collection, the latter continues for as long as the analysis of the material continues to provide new information useful for exploring the topic. Sample size in qualitative research is not defined in advance. It is determined by data saturation, usually defined as when the analysis of new material no longer yields new findings [64]. Saturation is, as Morse stated, “the key to excellent qualitative work” [65]. It is indeed an essential criterion of validity in qualitative research, especially for qualitative studies intended to lead to PRO development [66], as it ensures in-depth study of the phenomenon and suggests that further interviews are unlikely to produce new findings. This point has been heavily criticized, however, for it appears impossible to affirm saturation with certainty; that is, even if data saturation is a helpful idea for qualitative researchers, there are no pragmatic and consensual guidelines for determining when the point of data saturation has been reached [67].

For this reason, in line with grounded theory approaches, we prefer the concept of “theoretical sufficiency” [68]: data collection and analysis are complete when the researchers consider that the axes of experience obtained provide a sufficient explanatory framework for the data collected. Our minimum sample size is always at least 20 subjects, a choice made to optimize the visibility of our work: it enables publication in specialized journals accustomed to the large sizes in quantitative samples and randomized clinical trials, and unfamiliar with qualitative research, where sample size is not a criterion of methodological rigor.

### Stage 4: data collection, access to experience

The quality of the data collection determines the quality of the results. The goal is to reach the narrative of the experience [69]. The tool used to obtain this narrative always depends on the context, in either individual interviews or focus groups. Researchers in charge of data collection procedure should always consider the risk that face-to-face questioning might limit the subjects’ ability to narrate — to talk about — a subject so deeply personal, especially vulnerable persons [70] and adapt the data collection process to ensure they reach this narrative. Patients can find it difficult, even intrusive, to talk about their lived experience of a disease [71]. Most of the time, the IPSE data collection procedure relies on visual narrative support for the participants, aimed at enhancing the narrative by reducing inhibitions. This support may be a photograph, or a clinical vignette, or a short video clip directly related to the experience under study. It also facilitates the relationship and communication between the researcher and the subjects [70].

Photo-elicitation is the visual narrative support method we use most often [72]. This tool helps participants to think about a picture — ideally one they took themselves or, sometimes chosen with caution by the research group for the purpose of introducing the object of the study without influencing the participants. The positive effects of photo-elicitation on the research process have been widely described in qualitative literature: it improves the quality of the data collected [73], it promotes active cognitive involvement and better participation in the research [74] and when the participants take the picture themselves, it empowers them, by putting them in a more active position and thereby giving them the opportunity to influence the research process more strongly [75].

In interviews, the qualitative researchers systematically start by asking the participants to comment and react to the experience-related visual support. The latter usually begin and spontaneously continue speaking about it and especially their own experience.

The researchers then move on to open-ended questions [76], structured by the areas to explore, developed in turn from: (i) reading two pilot interviews, which will not form any part of the data analyzed for the study, (ii) the qualitative researchers’ own thoughts, each with different insights according to his or her own explicated preconceptions on the subject, and (iii) the knowledge and representations of the physicians experts in the topic. The group collectively chooses the areas, but these may be modified throughout the research process by each interview conducted. The interviewers use an interactive conversational style [77]. In an IPSE study, participants are considered the experts on their own experience. Qualitative researchers must conduct interviews that offer them the opportunity to recount it. In practice, they use prompts based on the “life-world” [78], a phenomenological concept with five dimensions (i.e., lived body, lived time, lived space, otherness and selfhood) through which the everyday actions and thoughts of the participants can be explored. All interviews are recorded and then transcribed verbatim, including the nonverbal aspects (e.g., pauses, hesitation, and laughs). The data are anonymized. The transcripts obtained are the object of the analysis.

### Stage 5: data analysis, from the structure of the experience to its translation into concrete proposals

**Fig. 2:**
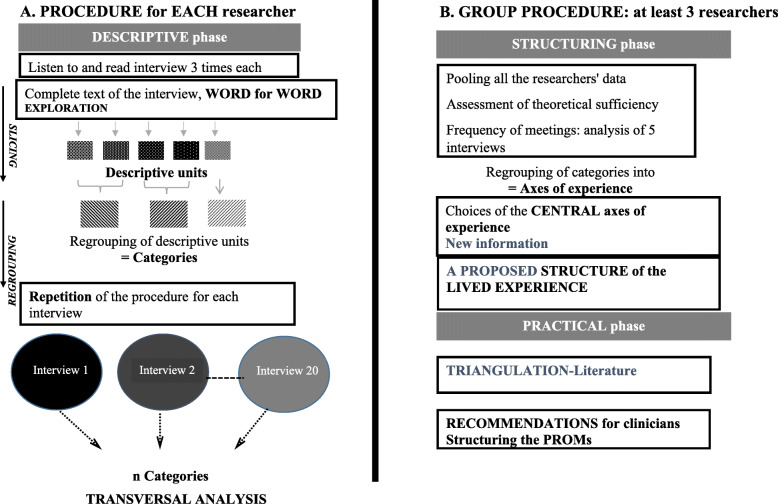
Stage 5 of IPSE. From the demonstration of the structure of the experience to its translation into concrete proposals. **a.** The procedure of each researcher, initially individually, corresponds to the descriptive analysis phase, including: i) listening to and reading the interview, ii) cutting up the text in descriptive units and then regrouping them into categories, This operation is performed for each of the 20 interviews, which are analyzed transversally. **b.** This structuring phase involves a group procedure (at least 3 researchers) with regular pooling of the data and analysis, during which the theoretical sufficiency is assessed. During this phase, the axes of experience are produced and the group determinates the central axes of experience, which result in the proposal of a structure of the lived experience. Finally the practical phase, which leads from triangulation by the literature to concrete proposals (guidelines, PRO)

Our analytic process is rigorous, detailed, systematic, and sharable. It relies on an inductive, phenomenological method and is intended to lead to concrete suggestions for improving aspects of treatment and of the health care pathway. It has two stages: one of independent work by individual researchers, and one of pooling the data collectively, by the group (Fig. 2).

#### Individual procedure - descriptive phase

At least three of the qualitative researchers independently and simultaneously conduct a systematic descriptive analysis aimed at conveying each participant’s experience. This procedure is not original; it has been already proposed in other phenomenological approaches, including IPA [15] and that Colaizzi’s method [40]. In fact, our descriptive analytic phase is inspired by Colaizzi’s analytical procedures and leads to the drafting of a structure of the experience, related to what Colaizzi named the “fundamental structure of the phenomenon” [79].

For each interview, this involves

- **Listening and reading**: we suggest that qualitative researchers analyzing the data listen to the recorded interview twice — once without taking notes, and a second time while taking notes throughout. They can refer to these notes during the analysis and share them (or not) during the group process. Next, they read the interview transcript three times, taking notes at each reading. This process bathes the researchers in each participant’s expressive style and enables an overview of the narrative. These numbers of times to listen to and read the interview are of course suggestions; these remain the decision of each researcher. For instance, some researchers prefer to transcribe their own data and do not need to listen and read as much as a researcher *discovering* the data for the first time. However, in our experience, for data not transcribed by the researcher, listening twice and reading three times appear to be a perfect compromise to really saturate oneself with the material without wasting too much time.
- **Exploring the experience word by word**: The researchers explore the interview meticulously and cut up the entire text (in units of one or several words) into segments called ***descriptive units*** (Table 1). These descriptive units are not pre-established and remain as close as possible to the participants’ words.
- **Regrouping the descriptive units into categories**: The units are categorized, that is, they are regrouped according to their proximity of meaning and experience, wherever they may be in the interview. This reorganization reveals the framework of the participants’ experience (Fig. 4).

**Table 1:** Word for word exploration, example of *découpage*

Transcript (excerpt)	Words to be coded	Descriptive units
*Interviewer: What exactly is it that is complicated?*		
**Woman: I have the impression that people look at my hands, they are strange, these hands...so I tend to hide them.**	I have the impression	Have the impression
	People are looking at my hands	Looking at my hands
	they are strange, these hands	Strange hands
	So I tend	Tend
	To hide them	Hide my hands
*Interviewer: I don’t understand, I’m sorry, you hide your hands because who is looking at them?*		
**Woman: The people at work look at my hands**	The people at work	colleagues
	look at my hand	Looking at my hands
*Interviewer: The people at work in general or some people in particular?*		
**Woman: Wait, I’m thinking, it’s more the women, finally I’m more often with women too, yes, it’s more the women**	Wait	Wait
	I’m thinking	thinking
	it’s more the women	more the women
	finally I’m more often with the women too	Environment of women
	it’s more the women, yes	More the women
*Interviewer: It’s different for you, that is, the gaze of another woman or of a man — you’re going to experience them differently?*		
**Woman: Yes, yes I’m realizing that now, but it’s totally crazy, it’s that the women looking at me is going to bother me more, is going to be more ...be more…**	Yes, yes I’m just now realizing it	Understand
	now	now
	but it’s completely crazy	ridiculous
	It’s that a woman looking at me	Women looking
	bother me more	be bothered
	is more … be more …	More the women
*Interviewer: More?*		
**Woman: I’m going to be more sensitive… but I don’t know why. You have the answer, because I don’t.**	I’m going to be more sensitive	Hesitation
	but I don’t know why	be more sensitive to it
	You have the response	Not know why
	because I don’t	You have the response
		Not know why
*Interviewer: No I don’t know either, you’re a woman yourself….*		
**Woman: I have always had the impression that women observe more than men… or else, yeah, I have the impression that the women stare at me much more than men do.**	I’ve always had the impression	Have the impression
	that women observe more than men…	Women observe more
	or else	Or else
	yeah I have the impression that	Have the impression
	the women stare at me much more than the men stare at me	More the women Be stared at

These stages are carried out with the help of “QSR” NVivo12 software to create and assemble the descriptive units and provide graphic support for their reorganization. This descriptive analysis is performed separately for each interview. Progressively, the researchers independently analyze all of the interviews thus far explored, cross-sectionally, by regrouping similar categories and excluding none of them.

#### Group process-the structuring phase and then the practical phase

During this group process phase, the three researchers who segmented and coded the text now meet and work with the other group members, that is, the physicians experts in the topic, the other qualitative researchers, and the patients, all of whom have familiarized themselves with the data (by listening and reading all the interviews as many times as necessary), without performing the descriptive analysis. The group’s heterogeneity has a heuristic function essential for the construction of the results: the group enables the co-construction by all group members of important points of the experience linking a set of implicit perspectives, made up of each researcher’s culture, theory, knowledge, sex, function, and background. The three researchers meet with the rest of the group after the analysis of five interviews, then 10, then 15, then 20, etc. … to share the categories that have been uncovered and assess their theoretical sufficiency. In practice, for organizational reasons mostly, group meetings can occur after the analysis of more or less than five interviews; however, we recommend this rhythm of meetings because of our experience: we found that sharing the content of the descriptive analysis of more than five interviews can result in a superficial sharing that impairs the quality and originality of the results, while more frequent meeting appeared to be time-consuming without providing richer data analysis.

##### The structuring phase

**Fig. 3:**
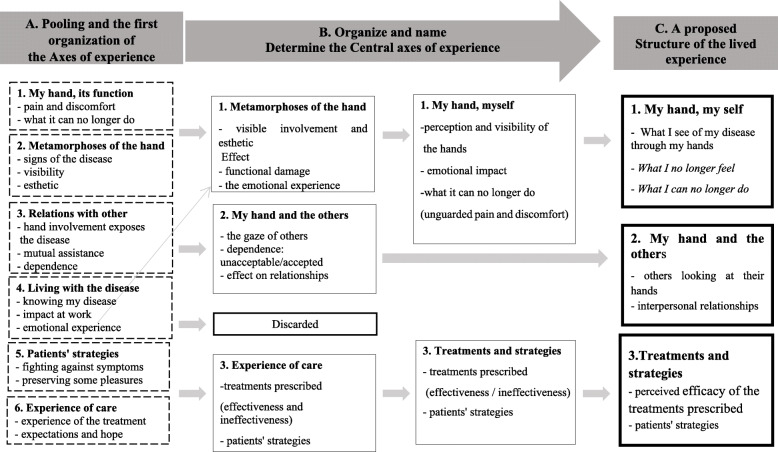
Intermediate stages of the structuring phase (from axes of experience to the structure of lived experience). The objective of the structuring phase is to produce a proposed structure of the lived experience. The three researchers meet with the rest of the research group. This collective group procedure can be defined as a co-construction by the researchers of important points of the experience. **a.** It involves sharing all the categories and constructing the organization of the axes of experience obtained during the descriptive phase, sometimes changing the name of the axes, sometimes changing categories from one axis of experience to the other. **b.** This is an intermediate stage of organizing or naming the axes. The structuring phase is a repeated act with an important dimension of choice. Some axes of experience will not discarded to determine the central axes of experience and terminate by **c.,** the proposed structure of the lived experience

In practice, during these two-hour meetings, the group must:

- **Regroup the categories into axes of experience:** The reorganization of the categories must uncover the framework of the participants’ experience, which we call its axes. These axes must be constructed such that each can be linked to its subjacent categories. Naming the axes must make it easy to read the results and highlight the original and relevant points. In practice, the names, the number, and the content of the axes may well change several times before their structure is finalized (Fig. 3).
- **Determine the structure of lived experience characterized by the central axes**: this is an action that is delicate, iterative. This phase involves an important dimension of choice. Exhaustive results, unranked, may dilute the original points and the new information, thus impeding any translation of the results into direct implications. The final structure of lived experience does not reflect the many intermediate stages required to reach it, stages during which some axes of experience will be regrouped and some even abandoned. It is very important during this phase that the physicians who analyzed the literature consider and discuss the originality and relevance of each axis, or on the contrary, its previous mentions or triviality according to the literature.

**Fig. 4:**
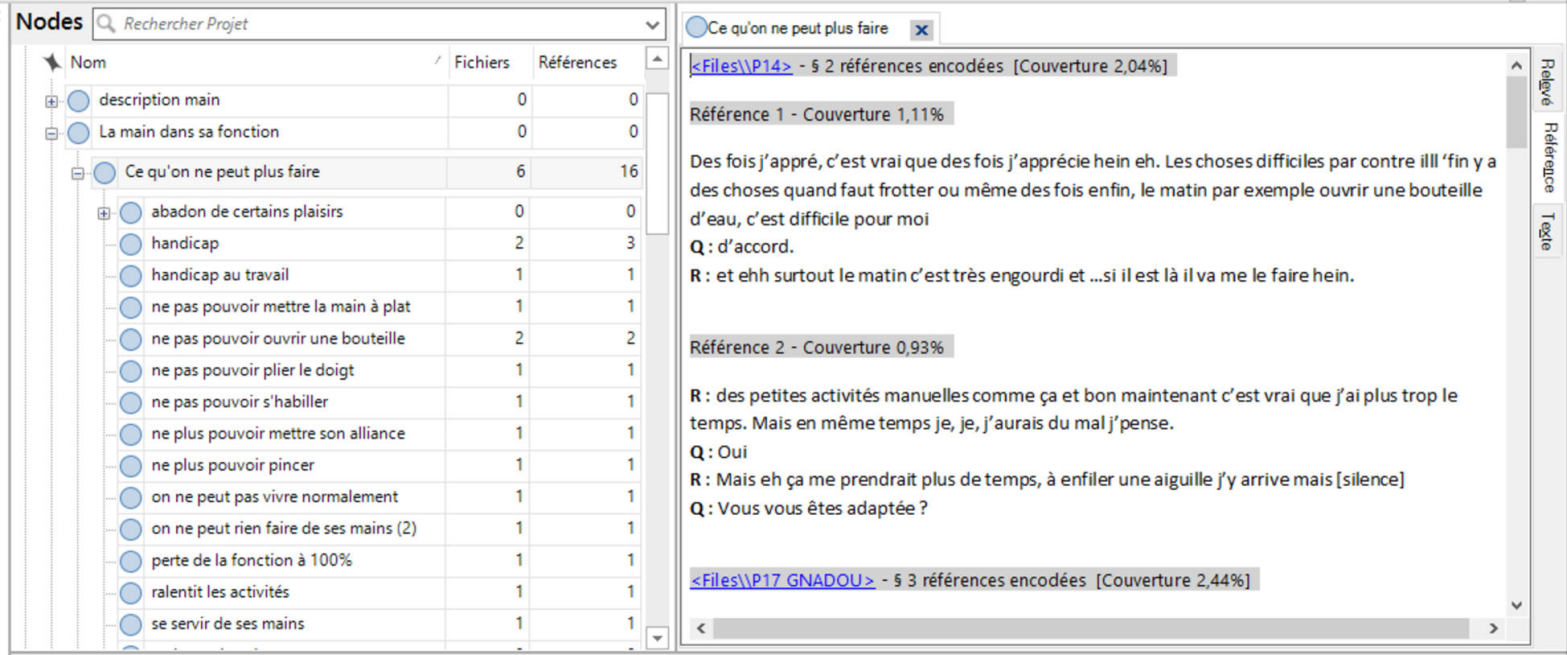
Regrouping of codes into categories with NVivo 12

At the end of these two stages, the group, all together, writes up a proposed structure of the experience within the context in which it was explored, composed of its central axes of experience; this will be the study’s results section.

##### Practical phase

The objective of this phase is the signature of the IPSE method, concordant with Thorne’s interpretive description [21]: the translation of the findings into proposals about the health care pathway, its clinical implications, and the perspectives for further research. Accordingly, the results will propose, for example, supports for interviews or practical recommendations that physicians can use directly with these patients.

A process of triangulation with the data in the literature completes the analytic process. This is not a process original to IPSE, of course, but rather a good practice that should be followed in all qualitative health research [20, 22]. The physicians experts in the topic and responsible for the literature review now share with the group their in-depth analysis of the literature from several databases (PubMed, PsycINFO, CINALH, and Web-of-Science (SSCI)) to identify the original aspects of the results and their differences and similarities with the literature.

The completed study is then reported in a scientific article that meets the COREQ criteria [80]. COREQ is a useful checklist of items that should always be included in reports of qualitative research. Although some items are directly related to criteria for analytic rigor, such as reflexivity or triangulation, the COREQ checklist provides an outline for reporting important aspects of qualitative research and not guidelines for performing it.

### Rigor and methodological quality of IPSE

We identified and operationalized seven methodological points to ensure the quality and rigor of IPSE studies. Some are already recognized criteria of rigor for qualitative research (triangulation, attention to negative cases, transferability, reflexivity), others are innovative and specific:

#### Patient involvement and feedback of the “subjects of experience”

We have already mentioned that some patients should be part of the research group, or at least be invited to help develop the research question and to give feedback about the results. For the latter point, many authors, including Colaizzi, recommend that participants validate these results. There are practical difficulties in obtaining feedback from the entire group of participants. Our method offers a methodological innovation by progressively replacing feedback by study participants by presentations of the study and conversations with other subjects of the experience. This action ensures the credibility of the results and guarantees their transferability. The results obtained by the IPSE method have a singular status, as accessible and expected, but also uncovered and surprising. The expected effects are both agreement and surprise, through the uncovering of evidence “hidden” until then. The subject of the experience should be able to say: “***it’s exactly that, but I had never formulated it like that*”.**

#### Triangulation

This concept refers to the use of multiple methods or data sources as a rigorous procedure to ensure a global understanding of the phenomenon under study [81]. There are four types of triangulation: “method triangulation, investigator triangulation, theory triangulation, and data source triangulation” [82]. In an IPSE study, at least three researchers are involved with data collection and individual analytical procedures; several data collection techniques can be used in the same study (for instance, individual interview and focus group); and triangulation with the literature is systematically carried out.

#### Attention to negative cases

Particular attention must be paid to these cases in which new elements can differ radically from the emerging structure of the experience. Most of the time, these negative- sometimes contradictory- cases will be integrated into the results. If a case differs completely from the proposed structure of the experience, we consider that theoretical sufficiency has not been reached and conduct new interviews and analyses.

#### The question of the choices of the central axes of experience

Our objective is not knowledge for its own sake, but knowledge for improvement in patients’ care and in their lives. The choices, always guided by this objective, are determined by the relevance and not the recurrence of the axes of experience.

#### Researchers’ subjectivity and reflexivity

The issue of reflexivity must be addressed. It can be defined as the researchers’ reflection of their role in the study and its effects on their findings at every step of the research process [56]. A recurrent hazard in qualitative research is that the results become the reflection or confirmation of the researcher’s preconceptions and beliefs [83]. The process of reflexivity enables researchers to avoid the pitfalls of applying their own preconceptions and assumptions to the material. They must take care to clarify their position, as much in their encounter with the material to be analyzed as in the research group meetings. To do this, the researchers involved in the study must answer these two questions regarding the study:

- ***(i) What are my preconceptions and my beliefs about the phenomenon under study and the research question*?** To address this question, they must each list all of their preconceptions and beliefs first to themselves and then share them with the group
- ***(ii) What are my expectations regarding this study?*** The researchers must question — themselves and each other — their personal motives to be a part of the research and what they are expecting to find or to achieve.

This reflexive position is worked on constantly in the group, during open discussions between the researchers. More than in the field notes, it is in the conversations, exchanges, and discussions between the researchers that reflexivity accomplishes its work.

#### Transferability

Qualitative research studies are performed in specific contexts. What matters for their results however is that they are transposable [84]; in our IPSE approach, this means that the structure of the experience is transferable, that it resonates with what other patients live beyond the context of the study. The assessment of the transferability of results ultimately lies with readers, who must decide if the setting of the study is sufficiently similar for its results to be transferable to their own context [85]. We ensure the transferability of the structure of the experience by obtaining feedback from patient associations or other representative groups. Also, as shown by the creation of a PRO tool validated by a quantitative psychometric study (currently being written up) in scleroderma, validation in a large sample of other subjects demonstrates the transferability of our results. A tool developed and structured by our method focusing on the lived experience of patients appears transferable and closer to reality than tools based on the theories and inferences of the professionals who create them [86].

#### The language of the analysis

It seems important to specify that our position is to anchor our research work in the participants’ language as well as their words. The research is conducted entirely in French, the language of the participants, and the researchers develop and write the results in this same language. Finally, at the last stage, the article is sent to a bilingual (English-French) professional scientific translator, and the authors and translator consult frequently to ensure that the words and meaning stay as close as possible to those of the participants.

## Results

The use of the IPSE approach has provided original findings enabling practical implications, such as (1) development of PROs focusing on areas not yet covered by existing scales, (2) clinical recommendations concerning assessment and treatment, (3) innovative ways to improve communication between patients and doctors, and (4) new insights for medical pedagogy.

### PRO development

#### The lived experience of hand involvement in patients with systemic sclerosis (SSc)

Table 2 presents an exemplary IPSE study exploring hand involvement among patients with SSc. The structure of lived experience described in our results revealed that the distress of patients dealing with functional impairment of their hands is linked especially to the loss of what had been important parts of their lives before the disease (leisure activities and hobbies, work, a musical instrument, a family activity). In other words, the intensity of the functional impact was related to “what I can no longer do” rather than to “what I cannot do.” The existing scales either focus on the very targeted assessment of individual components of hand involvement in SSc (the *Raynaud’s Conditions Score* [87], the *Hand Mobility in Scleroderma Scale* [88], and the *Delta Finger to Palm* [89]) or are generic functional scales validated for this disease, evaluating the functional impact of this involvement and its daily repercussions, at home, at work, and on QoL (the *Cochin Hand Function Scale* [90], the *Arthritis Hand Function Test* [91], and some specific items of QoL scales for scleroderma, such as the *Scleroderma Health Assessment Questionnaire* [92]). These scales evaluate the ability to perform some actions of daily life, such as cleaning or getting dressed, but do not include this dimension of a function they once had, which had been important, and was now lost. Here, access to the lived experience and its rigorous analysis make it possible to show that it is this loss of function that is painful. Similarly, our results show that patients develop strategies to compensate for this functional involvement, which no longer presents a problem in their daily life, although functional scales continue to detect it as a functional problem.

**Table 2:** Example of an IPSE study: the lived experience of hand involvement in patients with systemic sclerosis (SSc)

Context	SSc is a rare autoimmune chronic disease characterized by vascular injury, immune dysfunction, and an abnormal fibrotic process that can affect multiple organ systems including the skin, lungs, gastrointestinal tract, and cardiovascular system. Skin is always involved, sometimes with Raynaud syndrome and other frequent forms of hand involvement, associated with various often cumulative processes: joint, cutaneous, microvascular, bone, and nerve abnormalities. The repercussions of hand involvement play a central role in functional limitations and affect quality of life substantially. Existing quantitative research on hand involvement in SSc has mainly evaluated its functional, disabling, and esthetic aspects. These studies are based on preconceptions of how to define and measure hand involvement, preconceptions that do not come from patients’ lived experience. No published research has provided patients with the opportunity to describe their experiences and perspectives about the emotional challenges and areas of distress they face.
Objective	To explore how patients with SSc experience the effect of their disease on their hands
Stage 1	Research group included - two dermatologists specialized in SSc: LV, a professor, and DH, a resident in this specialty; - three researchers expert in qualitative methods (JS, a male psychiatrist, ARL, a female professor of medicine and EM, a female psychologist). The group’s members were highly diverse especially in terms of knowledge, age, and background. The group started the research by meeting a group of patients (*N* = 5) recruited from LV outpatient practice, in the Caen University Hospital. All of them emphasized the central role of hand involvement in their daily lives.
Stage 2	The two clinicians reviewed the qualitative and quantitative literature systematically to confirm the relevance and originality of the study. They verified that no qualitative study had dealt with the specific experience of hand involvement in SSc, although a substantial qualitative literature has looked at the global experience of SSc patients as a whole. To maintain an inductive process, the other group members did not have access to this review until the practical phase of Stage 5.
Stage 3	The research group defined the inclusion and exclusion criteria, intended to attain this exemplarity. Inclusion criteria: - Age: 18 or older - Scleroderma diagnosis according to the ACR/EULAR criteria (European League Against Rheumatism, 2013) - Involvement of the hand (Raynaud phenomenon) - Agrees to participate in the research Exclusion criteria: - Age < 18 - Psychiatric disorders or impairments of cognitive function that would prevent a useful interview The group selected an appropriate study site: a multidisciplinary outpatient department, in the Caen University Hospital, where dermatologists, rheumatologists, vascular specialists, rehabilitation specialists, orthopedists, and internists assess and treat patients with SSc, especially those with hand involvement. The University of Paris-Descartes *Council for Health Research Ethics Assessment* approved the study protocol. All patients provided informed written consent before inclusion. Because patients had several consultations a month, a large amount of clinical and paracliniclal data were available: clinical elements, psychometric assessments, and additional examinations. Social and demographic data, Rodnan scores (of skin thickening), the Cochin Hand Function Scale (measuring functional involvement of the hand), and scores on the specific Scleroderma Health Assessment Questionnaire (SHAQ) QoL scale were collected for all included patients. These data were not to be analyzed but collected to present the clinical characteristics of the sample in detail. We used our purposive sampling strategy and included patients differing by age, social and family status, hand involvement, disease history, and comorbidities, continuously looking for potential heterogeneity. Data saturation was reached with 12 patients, but to ensure the theoretical sufficiency of our results, we included 21, reaching our preferred minimum sample size of 20 participants.
Stage 4	A researcher first met each participant to obtain his or her written consent and to collect social and demographic data and any relevant clinical scores. This facilitated the subsequent research interview, a few days later. It was clear that data for this study had to be collected in individual interviews as we wanted to reach an individual lived experience. We were nonetheless aware that patients might find it difficult, even intrusive, to talk about parts of their sick bodies. We therefore chose to provide them with visual narrative support: a photograph of a hand with Raynaud’s phenomenon, a characteristic disabling manifestation in 95% of SSc cases (Fig. 4). Photo-elicitation has not previously been used in any qualitative study of patients with SSc. The interview systematically began by asking participants for their reaction to the photograph. We conducted open-ended interviews, structured by areas to explore, chosen collectively by the group. These areas were selected after a review of two pilot interviews based solely on photo-elicitation: 1. Disease history, onset of the disease (potential question: How long have you had this disorder?) 2. Hand involvement (potential question: Can you tell me how your hands are damaged by this disease?) 3. Daily life (potential question: What bothers you the most on a daily basis?) 4. Emotion (potential question: How does it make you feel when you think about the current state of your hands?) 5. Care (potential question: What was helpful?) Each interview lasted from 45 to 60 min. They were conducted by two experienced researchers, from February 2015 through April 2016.
Participants	This study included 21 participants: 18 women and 3 men. Their mean age was 60 years at the time of the interviews. 18 had limited cutaneous SSc and 3 diffuse cutaneous SSc. Its effect on their hands were diverse: all had Raynaud syndrome (*n* = 21), but some also presented joint involvement (*n* = 10), calcifications (*n* = 11), sclerosis (*n* = 15), telangiectases (*n* = 6), hyperkeratosis (*n* = 6), digital ulcers (*n* = 12), digital necrosis (*n* = 1), and sub/periungual hemorrhages (*n* = 6). The mean Rodman score was 8.1/51, the mean score on the Cochin Hand Function Scale was 15.6/85, and the mean SHAQ score 1.2/3.
Stage 5 Structure of Experience	The analysis of the interviews enabled us to identify three central axes of experience: (1) my hands, myself; (2) my hands and others; and (3) treatments and strategies used by the participants. 1. **My hands, myself** *(i) What I see of my disease through my hands:* The patients considered the involvement of their hands was visible. They reported changes in the color, shape, and texture of their fingers, most often evoking emotions of rejection or disgust. Many described a major esthetic impact, saying their hands are ugly to look at, again with a strong emotional component. Finally, and especially, the participants considered this visible hand involvement as evidence of their disease and its prognosis, experiencing this damage as a permanent reminder of their illness and a witness of its development and its severity *(ii) What I no longer feel:* Most participants complained about the loss of sensitivity in their hands. They reported numbness, a feeling of skin tightness, or constantly cold hands. They underlined the daily effect of this loss of sensitivity. Numerous patients also complained about hand pain that handicapped them in their daily lives. (iii) *What I can no longer do:* The participants underlined what they could no longer do because of the impairment of their hands. All mentioned an impact on their work. Depending on their job, some felt helpless, unable to perform their work duties. Others found it impossible to keep their jobs. Some explained that they continued working, despite the pain. Finally, others directly linked the hand involvement to their difficulties in finding employment. Participants also reported major problems doing housework. Finally, many reported having had to give up hobbies or pleasures that mattered greatly to them, such as gardening, sewing, or playing music. The functional impact was always associated with emotions of anger, frustration, or sadness. **2. My hands and others** *(i) Others looking at their hands:* Most patients considered that this hand involvement exposed their disease to the eyes of others. Some stressed the impossibility of hiding their hands from the sight of others; others mentioned esthetic discomfort that was greatest in social interactions and starting the instant they observed someone looking at their hands. They inferred the negative thoughts of others who looked at their hands. They imagined that these people had to find their hands strange or “dirty” and would worry about the potential risk of contagion. *(ii) Interpersonal relationships:* All participants reported an important impact on relationships, especially in interactions with those close to them. Many complained that they had to ask others for help every day because of their hand impairments. They reported experiencing unbearable dependence, more unbearable for some than the disease itself. They also reported a feeling of that they could no longer do these things themselves. Some said they accepted help only out of a feeling of resignation, while others preferred to refuse the assistance offered, even if they suffered. Less often, some patients said they were aware of their disability and had no trouble asking for help with some activities. These aspects of assistance and dependence were especially present in the participants’ descriptions of their relationships with those close to them. Some underlined essential support from their family, while others grumbled that their actions and behavior were constantly watched. Some participants found their family’s support inadequate and reported conflicts with them; they felt lonely and misunderstood facing a life encircled by restrictions. Finally, some reported especially what they could no longer do with those they were close to, special moments they could no longer share. Only two mentioned in this regard an effect of their hand impairment on their sex life. **3. Treatments and strategies** *(i)Perceived efficacy of the treatments prescribed:* Many patients reported the direct efficacy of some drugs that made their symptoms disappear; nonetheless, they regretted the transience of their effect or that they could not use them more often. Similarly, surgical treatments were described as effective, helping to relieve the pain. They were sensitive to techniques that permitted functional improvements and judged their efficacy indirectly, by the lack of disease progression. Sometimes they found that neither medication nor physical therapy was effective. Others, however, considered that the treatments’ adverse effects outweighed their positive effects. *(ii)Patients’ strategies:* the participants reported the strategies that they used to battle hand damage on a daily basis and to preserve some pleasures. They had developed various means, most often material, to overcome these impairments: using a dishtowel to open a bottle, a nutcracker or a screwdriver to open jars, pliers to grasp small objects, etc.). All explained that they wore gloves, in summer as in winter, to prevent Raynaud’s phenomenon. Some complained about the discomfort these gloves engendered. They also described their strategies for maintaining some pleasures, by finding ways to preserve or adapt their hobbies and leisure activities.
Stage 5 Practical Phase	The structure of lived experience has revealed that the intensity of the functional impact was related to “what I can no longer do” rather than to “what I cannot do.” The question of the **visibility of their hand impairment,** to themselves and to others, was crucial, with two original aspects revealed by our study: i) the permanent exposure of their disease to the eyes of others — both in social interactions and in more personal relationships — underlines its effect on relationships; ii) it serves as a permanent reminder of the disease to the patients, inducing constant concerns about their survival, their existence. This revealed the importance of other dimensions than functional impairment in the lived experience: **esthetic, relational, emotional and existential**. Practical implications for healthcare professionals: - PRO development: construction of the **HaNDE scale**, - Recommendations for clinicians: Clinicians should routinely evaluate hand functions that patients used in ways important to them and have now lost, the impact of the visibility of the disease due to this hand involvement, and should also explore the esthetic, emotional, relational, and existential issues that result. Our results also suggest the need for trust in the patient/physician relationship, to alleviate the patient’s distress in dimensions inaccessible to medication.
Criteria of Rigor	Feedback to subjects of experience: In the study presented in this paper, we used all of these criteria to ensure the rigor of the analysis and thus the trustworthiness of the results. The feedback of “subjects of the experience” was conducted by presenting the research to a group of patients (*N* = 20) from the department of internal medicine at Cochin Hospital, a reference center for SSc. These patients all recognized their own experience in the structure we proposed. The question of the choices of the central axis of experience: only two participants mentioned the effect of hand involvement on sexual relations. We included this subject in our results to improve the patients’ lives, because this subject is difficult for patients to raise with doctors. Triangulation, reflexivity, attention to negative cases
Limitations	1. It took place in France, and caution is required in transposing our results to other places because medical care depends strongly on the organization of the medical system as well as on the country’s economy. 2. This was a single-center study and all patients recruited in this study were followed in specialized departments (dermatology, rheumatology, internal medicine, and others). It would be interesting to see the results of other studies reproducing this design in other medical settings, such as, for example, that of general medicine. However, we think that this study meets the aim of qualitative research: it may be transferable to other contexts.

The question of the visibility of their hand impairment, to themselves and to others, was also crucial in the lived experience of the participants. The esthetic repercussions on QoL in SSc have been already explored by dermatology QoL scales, the *Dermatology Life Quality Index questionnaire* [93] or the *Satisfaction with Appearance* scale *(SWAP)* [94]; and the esthetic impact of hand involvement can be assessed by the 6-item *Brief SWAP,* validated for SSc [95]. The IPSE approach enabled the emergence of two original results about the visibility of hand involvement: i) the permanent exposure of their disease to the eyes of others — both in social interactions and in more personal relationships — underlines its effect on relationships; ii) it serves as a permanent reminder of the disease to the patients, inducing constant concerns about their survival, their existence. Beyond the functional impact of hand involvement in SSc and the esthetic repercussions, relational and existential aspects directly associated with patients’ emotional distress also appeared to be an important part of patients’ experience. The different qualitative studies exploring the sources of emotional distress among people living with SSc [96–103] have never explored the lived experience of hand involvement in this disease. These emotional, relational, and existential aspects have never been described specifically for hand involvement in either SSc or other autoimmune diseases affecting the hands, and no scale used to assess hand involvement contains items assessing these aspects.

The IPSE approach has thus made it possible to enrich the data available on the lived experience of SSc patients with hand involvement. The current scales, obtained from questionnaires constructed without exposure to patients’ lived experience, inform clinicians especially about the functional dimension of their patients’ disease but do not allow them to provide comprehensive management for these patients that covers what matters from the patients’ perspective. It is especially important to take their perspective into account in that this is a chronic disease that cannot be cured and has no specific treatment. Our results thus allowed us to construct an appropriate, relevant –and missing- PRO tool, the HAnDE scale, intended to be a useful tool enabling detection of the different dimensions of hand involvement in SSc: functional (and loss of function), emotional, relational, existential, and esthetic. Based on our results and using the vocabulary of the patients we interviewed, we generated 18 items associated with their lived experience. A final version of 16 items was subsequently obtained during a validation study with 105 patients that showed the relevance of the scale for assessing the global experience of hand involvement in patients with SSc. This new PRO scale will be considered as an outcome measure in future trials. This illustrates the direct integration of IPSE studies in EBM.

#### PRO development in the field of child and adolescent psychiatry

Pediatric PRO assessment is a very recent field of research, and empirical evidence about quantitative instruments within this age-specific population is still scarce [104]. We are currently constructing two adolescent psychiatry PRO scales based on the results of two qualitative studies: (i) one about the treatment of adolescents with anxiety-based school refusal [105], and (ii) another assessing the therapeutic alliance in the treatment of adolescents with anorexia nervosa [106].

(i). This qualitative study, exploring how 20 adolescents with anxiety-based school refusal and 21 of their parents experienced psychiatric treatment, revealed some divergences between the two groups on their perception of efficacy and enabled us to construct a relevant PRO for both adolescents and their parents. This tool integrates original aspects found in our study: assessment of both external (*return to school*) and internal (*self-transformation*) goals of care, the duration of care (an effective treatment as rapidly as possible vs. the need for a treatment period sufficiently long to allow adolescents to change and develop) and unexpected care-linked relationships.
(ii). This study explored the experience of therapeutic alliance among 15 adolescents with anorexia nervosa (AN), 18 or their parents and their 8 psychiatrists. Crossing these three perspectives makes it possible to identify aspects that are missing in scales currently used to measure therapeutic alliance in the treatment of AN in adolescents, such as the AWAI or the HAQ-CP [107, 108]: parents’ negative representations of “psychiatry” focusing on somatic aspects of treatment and the omnipresence of the issue of relationships. Items on relationship in the scales currently used concern only the relationship with care providers and focus on its perceived quality. In our results, relationships are involved in all three components of the definition of therapeutic alliance: the quality of the association, the objective of treatment, and the means to achieve this objective. Similarly, the current scales do not mention the role of the adolescent–parent relationship, which appeared to play a key role in the construction of the therapeutic alliance in our results. We are therefore developing a PRO scale on this topic that includes specific items: (1) the quality of the relationship with staff as a means of getting better, (2) the impact on the alliance of parental involvement in treatment, (3) the impact of treatment on the parents’ point of view about the adolescent and the relationship between adolescents and their parents, and finally (4) agreement about the objectives for improving family relationships generally and adolescent–parent relationships in particular.

### Clinical recommendations

Clinical recommendations drawn from the results of IPSE studies are intended directly for the physicians themselves.

#### Assessment and diagnosis

Clinical implications from IPSE studies can be innovative concrete guidelines, supported by the structure of lived experience of patients and other stakeholders, to improve the quality of the clinical assessment and of the diagnosis process.

For instance, based on the results of the study of hand involvement in SSc, using the same rationale presented above, we provided clinicians with clinical implications regarding the assessment of hand involvement among patients with SSc: (i) clinicians must assess this involvement globally and not by segmenting the evaluation with several scales that target especially functional involvement; (ii) They should routinely evaluate hand functions that patients used in ways important to them and have now lost and the impact of the visibility of the disease due to this hand involvement, and (iii) should also explore the esthetic, emotional, relational, and existential issues that result.

In another study, we explored the experience of the diagnostic pathway among 20 patients with acromegaly, a rare disease with a substantial diagnostic delay. Our results revealed the direct associations between diagnostic delay and the doctor–patient encounter [28]. The literature has already emphasized the key role of any doctor, regardless of specialty, in identify the signs, symptoms, and comorbidities of acromegaly by becoming involved and *seeing the unseen*’ [109]. To identify the disease as early as possible, however, our results suggest that physicians must allow themselves to question the patient proactively and to consider clinical processes beyond their own specialty, in other words, *seeing the unseen* is not enough if physicians do not say the unsaid.

#### Therapeutic implications

The IPSE approach is particularly well suited to exploring complex therapeutic processes and the perceived efficacy of treatment. The approach enables the description of therapeutic levers and efficacy criteria directly relevant to patients and other stakeholders and could contribute to achieving a more person-centered medicine.

#### Improving patients’ lives

We conducted two studies to explore the lived experience of cancer treatment; one crossed the perspectives of patients (*N* = 30), their families (*N* = 30), and their oncologist (*N* = 10) [30], while the other one focused on what affects the quality of daily life of patients with cancer (*N* = 30) during active treatment [110]. Our results led to some clinical recommendations to achieve patient-centered cancer treatment, that is, that physicians integrate the dimension of care into the curative treatments performed so that patients to live as well and not simply as long as possible [30]. To achieve this task, we found an original therapeutic lever that acts like a relational tool for physicians: the support object, defined as an object, a relationship or an activity particularly invested by the patients in their daily lives, which makes them feel good and makes the cancer and its treatment bearable. When patients are able to choose and be involved with a support object, the physician must support them and converse with them on this topic to help them maintain this investment throughout the health care pathway and to establish a trusting relationship and therefore, according to our results, improve their quality of daily life, without using up very much of the physician’s time [110].

#### Improving families’ lives

Therapeutic implications drawn from IPSE studies can also concern families and relatives. For example, we conducted a study to explore how 20 older siblings describe and perceive the care received by their brother or sister in child psychiatric centers specialized in the management of children with Autism Spectrum Disorder [111]. The literature has already recognized the need for both global family-centered treatment approaches [112] and specific programs intended for the siblings of children with serious diseases [113] to help them cope with their brother/sister’s condition, but our study revealed that when older siblings play and claim a role of helping and caring for the child with ASD, they benefit from their empowerment and involvement in this treatment, and physicians benefit from their perspective on this treatment.

#### Recommendations addressing the care pathway

For the study of anxiety-based school refusal mentioned above [105], we were able to provide recommendations about the outcomes and the duration of care: treatment must last long enough, in a place dedicated to care, to allow adolescents to become involved in their care and to reflect on the personal changes they need, but also to offer them the possibility of multiple human encounters, some of which — expected or unexpected — will turn out to be determinant in their development. Treatment should strive to combine and coordinate two outcomes of equal importance: a rapid return to school for the parents, and a sufficiently long time in care to enable a self-transformation for the adolescents.

### Patient-physician communication

#### Using the structure of lived experience

First of all, the systematic undervaluation of symptoms by physicians reveals some distortion in physician-patient communication [114–116]. The integration of patients’ points of view into their management through the intermediary of structures of experience obtained with IPSE would promote this communication. In an ideal context of shared medical decision-making, the involvement of patients in their management requires that they receive complete and appropriate information on which they can base their choices. Complete information requires that they have been heard and that their narrative of their disease has been considered. In our study of hand involvement in SSc, we systematically provided feedback to the clinicians, so that they could apply these new results uncovered by our exploration.

#### Improving communication, reducing confusion

The IPSE approach, especially when crossing perspectives, can also provide innovative ways to improve communication between patients and doctors.

We conducted a study among 20 patients and 10 physicians aimed at exploring the experience of neuroendocrine tumors (NETs), rare gastrointestinal tumors characterized by their rarity, the difficulty of their diagnosis, their often better prognosis, and their complex and long management [29]. The primary — and original — result of this study is the important experience of confusion found among patients. We have provided a statement that all physicians can use to support patients diagnosed with NETs to reduce their confusion, especially the semantic confusion as it explicitly uses the term cancer. This communicative tool meets patients’ needs (i.e. silent symptomatology, name, evolution, treatment and monitoring) including the need to improve patient-physician communication. It has been used in specialized medical consultations but also in training sessions of medical trainees in oncology and gastroenterology.

### Medical pedagogy

Here again, the structure of experience can always directly serve as training support to provide to medical students with relevant information about how patients experience both their disease and their care. IPSE approach can also reveal specific needs or gaps in the physician’s training and provide new insights.

#### Revealing training needs

In our study crossing perspectives between patients with cancer, their families, and their oncologists, we found that physicians had difficulties dealing with patients’ negative emotions during consultations and that this could be a barrier to their access to the factors that improve the patients’ capacity to live as well as possible. We suggested that physicians dealing on a regular basis with patients with cancer should receive a specific medical education that directly addresses the issues of coping with, recognizing, eliciting and using patients’ feelings as a therapeutic tool.

#### New insights for medical pedagogy

In our study regarding acromegaly diagnosis, patients reported having faced deficiencies in the medical world’s awareness of acromegaly. Indeed, acromegaly is a rare disease that doctors see very rarely and are therefore unlikely to think about. Our results led to suggesting the intervention of patient experts [2] in medical schools, so students can hear about their experience of diagnostic errors that lead to diagnostic delay and about its early and current clinical signs. Future doctors, who have received such training, will be more aware of the need for a high level of suspicion and active questioning to reach a diagnosis and should thus be more likely to think of the signs observed or reported as potential indicators of acromegaly.

## Discussion

All the studies presented here, and their practical implications, focus on the day-to-day clinical practice of physicians: relationships and communication in care, duration of care, therapeutic alliance, care issues, and outcomes. These aspects, related to the patient’s subjectivity and the patient-physician relationship, are very often forgotten or even excluded from medical research [20]. We consider, along with other scholars in qualitative health research [20, 21], that many advances in medicine and patient care are impossible until qualitative methods are fully integrated into the research arsenal. Qualitative research should — but does not yet —have a major role to play in clinical medical research; use of these methods remains a minority, even marginalized, option. Many medical researchers still apply a hierarchy — based on a paradigm confusion — between research methods according to the sole presence of quantitative research criteria, such as sample size, objectivity, and reproducibility; they inaccurately conclude that qualitative research is inferior and reduce it to a secondary role, always conceived of in the context of mixed-method research [117]. In other words, qualitative medical research is a victim of the *burden of proof* [118] and of the *tyranny of the average* [119].

For qualitative research to be able to fully contribute to medical research, it requires better recognition and appreciation from the entire medical community. This is what the IPSE approach is trying to achieve by staking out a medical position within a rigorous and systematic qualitative method.

1. The IPSE approach is to be integrated within EBM through mixed-method study designs resulted from a pragmatic and mutually enriching partnership between qualitative and quantitative methods [120].
2. Setting up research groups involving both physicians and patients is an innovative and original idea. This multiples the perspectives and enriches the data and results. Moreover, patient involvement helps to direct research towards person-centered medicine and finally, it allows the research process to maintain an inductive approach providing new results while remaining anchored in relevant medical issues.
3. Almost everyone agrees that it is important to understand what patients and other caregivers are going through. But to what extent? The first objective of an IPSE study should always be to achieve concrete improvements in patients’ lives (or those of other stakeholders) by leading to practical changes such as PRO development and being used to construct health recommendations or policies, while respecting the fundamental principles of qualitative research.

However, the ISPE approach has some pitfalls. First, it is a very demanding and ambitious research method. An IPSE study is as constraining in terms of workload and time as any other clinical medical research. It also requires abandoning the idea that a qualitative research requires fewer human, financial, and technical means. Second, in exploring an experience in depth in an interview, the researcher can expose the subject’s distress, especially when the question concerns the experience of a disease. This is an important ethical point: the researcher must systematically report this distress to the patient’s physician.

## Conclusion

IPSE is an innovative method and an important contribution to current methodological developments aimed at improving the quality and rigor of qualitative research in the medical field, for it anchors the research to the lived experience of those involved in medical care (patients, family, professionals) and it proposes concrete suggestions based on the results, including the development of PROs. No structured qualitative methods have previously recommended the direct involvement of patient experience into PRO construction, before assessing psychometrics characteristics of the scale. IPSE is a qualitative method specific for clinical research in medicine, designed to enable the implementation of pragmatic improvements. Our approach, which allocates to the experience of all the stakeholders in medical care its necessary role in the research process, is part of the movement for collaborative person-focused medicine and can be integrated easily in mixed-methods study designs [1].

## Data Availability

The datasets analyzed during the current study are available from the corresponding author on reasonable request.

## Abbreviations

COREQ: Consolidated criteria for reporting qualitative research
EBM: Evidence-based medicine;
HAnDE: *Hand AutoimmuNe Disease lived Experience*
IPA: Interpretative phenomenological analysis
IPSE: Inductive Process to Analyze the Structure of Lived Experience
PPI: Patient and public involvement
PRO: Patient Reported Outcome
QoL: Quality of life
SHAQ: Scleroderma Health Assessment Questionnaire
SSc: Systemic sclerosis
